# Ultrasound-Guided Cannulation of Arteriovenous Fistulas in Hemodialysis Units: A Systematic Review

**DOI:** 10.3390/healthcare14213577

**Published:** 2026-10-22

**Authors:** Mario Martinez-Torija, Pedro Manuel Rodríguez-Muñoz, Nayara Roldan-Martinez, Cristina Rivera-Picón, José Alberto Laredo-Aguilera, Inmaculada Muñoz-Martinez, Ana Quesado, Alexandre Rodrigues, Juan Manuel Carmona-Torres, Víctor Serrano-Fernández

**Affiliations:** 1 Unidad de Hemodialisis, Hospital Universitario de Toledo, 45071 Toledo, Spain; mario.martinez@uclm.es (M.M.-T.); nayararm97@gmail.com (N.R.-M.); inmammenf@outlook.es (I.M.-M.); 2 Grupo de Fisiopatología y Medicina Regenerativa, Instituto de Investigación Sanitaria de Castilla la Mancha, 45071 Toledo, Spain; 3 Facultad de Fisioterapia y Enfermería, Universidad de Castilla-La Mancha, 45071 Toledo, Spainjuanmanuel.carmona@uclm.es (J.M.C.-T.); 4 Grupo de Investigación Multidisciplinar en Cuidados (IMCU), Universidad de Castilla-La Mancha, 45071 Toledo, Spain; victor.serrano@uam.es; 5 Faculty of Health Sciences, University of Castilla-La Mancha, 45600 Talavera de la Reina, Spain; cristina.rivera@uclm.es; 6 Escola Superior de Saúde, Universidade de Aveiro, 3810-193 Aveiro, Portugal; anaquesado@ua.pt (A.Q.); alexandre.rodrigues@ua.pt (A.R.); 7 Departamento de Enfermería, Facultad de Medicina, Universidad Autonoma de Madrid, 28049 Madrid, Spain

**Keywords:** hemodialysis, arteriovenous fistula, ultrasonography, ultrasound-guided cannulation, nursing care, pain

## Abstract

**Highlights:**

**What are the main findings?**

• Findings on cannulation success varied across trials, and the available evidence does not establish a consistent reduction in pain.
• Potential benefits should be considered alongside the longer procedure times reported in several trials.

**What are the implications of the main findings?**

• Structured ultrasound training and cannulation protocols may improve nursing management of arteriovenous fistulas in hemodialysis units.
• Larger, adequately powered randomized trials are needed to confirm effects on cannulation success, complications, satisfaction, and vascular access survival.

**Abstract:**

**Background/Objectives**: Arteriovenous fistulas are the preferred vascular access for hemodialysis, but difficult or unsuccessful cannulation may cause pain, complications, and access dysfunction. This review evaluated whether ultrasound-guided cannulation improves outcomes compared with standard cannulation. **Methods**: A systematic review was conducted according to PRISMA guidelines. PubMed, Web of Science, Scopus, CINAHL, Cochrane Library, and ClinicalTrials.gov were searched for randomized controlled trials comparing ultrasound-guided and standard arteriovenous fistula cannulation in hemodialysis patients. Findings were synthesized narratively. Risk of bias was assessed using RoB 2, and certainty of evidence using GRADE. **Results**: Of 1210 records identified, five randomized trials involving 142 patients were included. Some trials reported improved cannulation success or lower pain with ultrasound guidance, but findings varied across populations and outcome measures. Three of four trials reporting procedure time found longer durations with ultrasound guidance. A consistent reduction in complications or improvement in patient satisfaction was not established. Staff findings mainly concerned confidence and acceptability. The certainty of evidence was low. **Conclusions**: The effects of ultrasound-guided AVF cannulation on pain varied across trials. These uncertain findings should be interpreted alongside the longer procedure times reported in several trials. Larger randomized trials with clearly defined outcomes and analyses accounting for repeated observations are needed before recommending routine use.

## 1. Introduction

Renal replacement therapy is the treatment used in patients with end-stage renal disease. Since many patients require hemodialysis (HD) [1], the clinical evaluation of vascular access (VA) is essential in the management of patients with chronic kidney disease for two main reasons: on the one hand, it is a key factor in receiving high-quality HD; on the other hand, the morbidity and mortality of patients depend on the efficiency of VA [2].

VA is the key to effective HD, since without functional access, treatment is not possible. VAs for HD can be classified into two groups: arteriovenous fistulas (AVFs) and central venous catheters (CVCs) [3]. The use of AVFs, either native or prosthetic, presents more favorable results than the use of CVCs in terms of lower mortality, a lower percentage of technical failures and fewer hospital admissions [4]. In turn, AVFs can be native or prosthetic, with the former being the most appropriate VA option for these patients. Its creation and maintenance have become more complicated in recent years because of the increase in the age of patients who start treatment with HD and the consequent increase in comorbidities, such as vascular problems, heart problems or diabetes, which have a known negative effect on the status of the patient’s vascular tree [5,6]. The correct functioning of an AVF is vital for the treatment of HD and represents a significant concern for the multidisciplinary team in charge of its evaluation [7]. Although the guidelines recommend the AVF as the VA of choice, complications related to cannulation of the access canal can lead to increased morbidity. In many cases, these complications can result in hospitalization, the need for complementary tests, and even loss of access [8].

Ultrasound-guided cannulation has been described as an approach to guide needle insertion into arteriovenous fistulas (AVFs) during hemodialysis [9].

Therefore, it is essential to optimize all the stages that contribute to the survival of the VA and reduce events that generate morbidity, which include creation, monitoring and surveillance to prevent adverse events such as stenosis, thrombosis and, ultimately, dysfunction of the VA [10,11]. Although the use of USG cannulation of AVF has advanced and been standardized, there are no known reviews that synthesize the available evidence.

Thus, the objective of this study was to analyze the scientific evidence available to compare the use of USG versus traditional puncture to evaluate and compare the differences in terms of pain, time spent, success rate, reduction in complications, and both patient and nursing staff satisfaction.

## 2. Materials and Methods

### 2.1. Study Design

This systematic review was conducted in accordance with the Cochrane Handbook for Systematic Reviews of Interventions and reported following the PRISMA 2020 statement [12,13]. Ethical approval and informed consent were not required, as this work consists of a systematic review of the literature. This revision was registered in PROSPERO with registration number CRD42024613025.

Comprehensive literature searches were performed across Pubmed, Web of Science, Scopus, ClinicalTrials, CINAHL and Cochrane Library databases.

### 2.2. Information Sources and Search Strategy

The searches were carried out with the objective of answering the question in the following format: Population, Intervention, Control and Outcome (PICO) (Table 1).

In this way, the research question posed is as follows: In patients with AVF for the treatment of HD, does the USG cannulation of their AVF present greater benefits than traditional puncture in terms of pain, time, complications, rates of success and increased satisfaction of the patient and nursing staff?

The searches were carried out between January and March 2026 and were updated on 7 October 2026. The search strategies used to answer the research question are shown in Table 2.

There were no restrictions on the date of publication. In addition, the bibliographies of the selected articles were reviewed in search of relevant studies.

### 2.3. Inclusion and Exclusion Criteria

The inclusion criteria used to select the studies that were part of the review were as follows: (1) randomized clinical trials (RCTs), (2) studies evaluating AVF cannulation during hemodialysis, regardless of the professional background of the operator, and (3) studies written in English or Spanish. Eligible interventions used real-time ultrasound guidance during needle insertion for AVF cannulation.

On the other hand, the exclusion criteria established for the selection of articles were as follows: (1) studies whose objective was the assessment or creation of the VA [14,15] and (2) studies related to vascular puncture of veins and arteries for other procedures, such as collection of blood or infusion of intravenous drugs.

### 2.4. Selection Process

**Figure 1:**
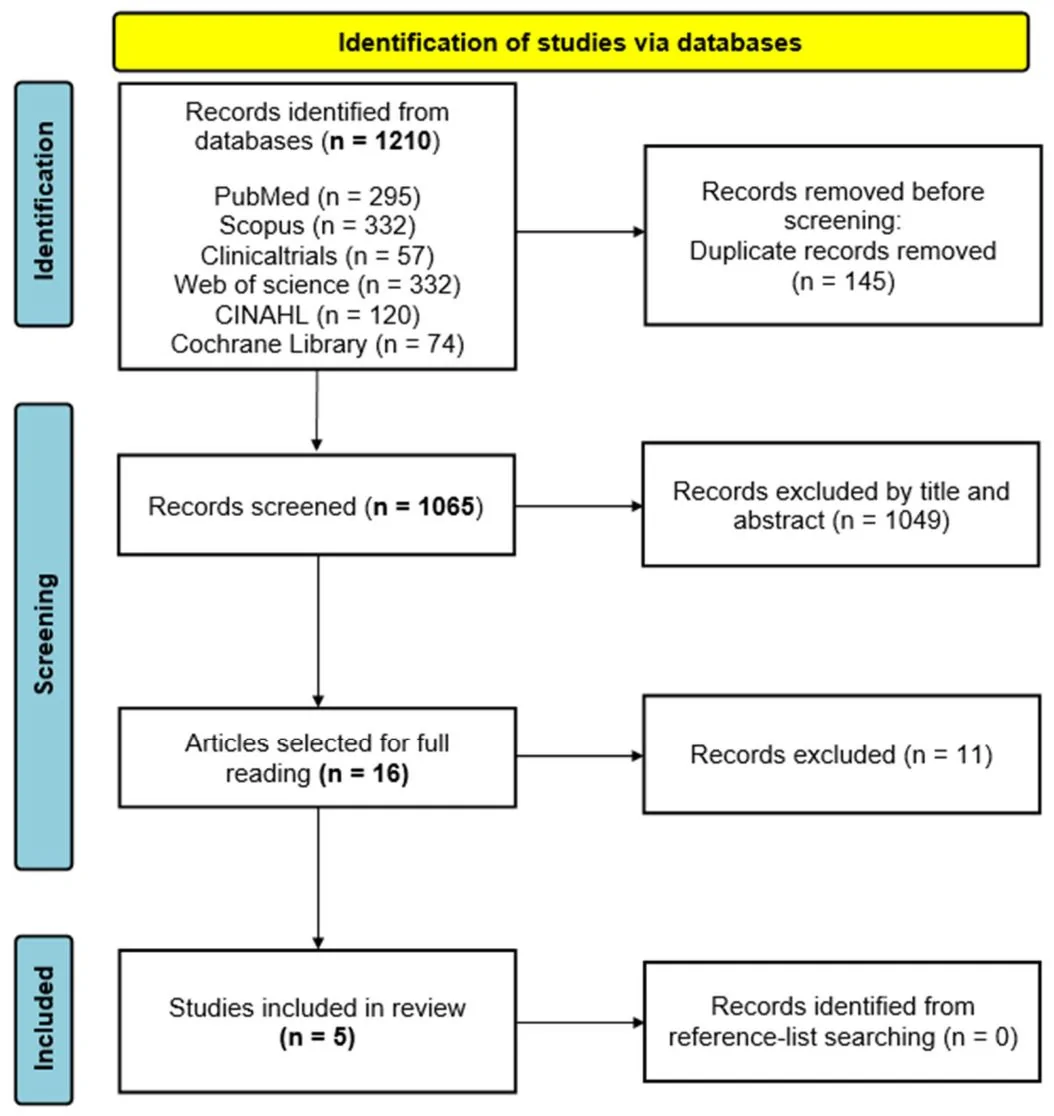
PRISMA flowchart.

The selection of studies was carried out by Mario Martinez-Torija and Víctor Serrano-Fernández For this purpose, the inclusion and exclusion criteria described above were considered. To manage citations and eliminate duplicates, the Mendeley bibliographic manager was used. After eliminating duplicates, the results were filtered by title and abstract to assess the studies suitable for their complete reading. After the complete reading, the studies included in the systematic review were selected. If an agreement was not reached, the authors Mario Martinez-Torija and Víctor Serrano-Fernández discussed it. In case of doubt or discrepancy, a third author (Juan Manuel Carmona-Torres) was consulted until an agreement was reached. The detailed selection process is shown in Figure 1.

Reports excluded after full-text assessment, together with the reasons for exclusion, are listed in Supplementary Table S1 [16,17,18,19,20,21,22,23,24,25,26].

### 2.5. Data Extraction

Data for the narrative synthesis were extracted jointly by two authors (Mario Martinez-Torija and Víctor Serrano-Fernández). In each case, the following data were collected: (1) authors, country and year; (2) study design; (3) study population; (4) intervention; (5) main results (decrease in pain, time spent, complications, success in cannulation and patient and nursing satisfaction); (6) conclusions; and (7) risk of bias.

### 2.6. Quality Assessment

**Figure 2:**
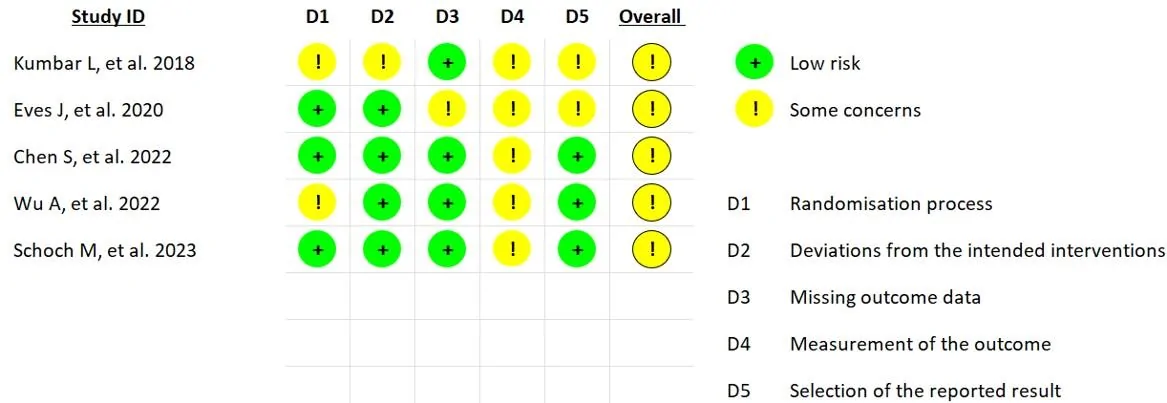
Risk of bias assessment of the included randomized controlled trials [28,29,30,31,32].

To assess the quality of the studies and the risk of bias, the RoB-2 tool for RCTs was used [27]. This tool consists of 5 domains: (1) the randomization process, (2) deviations from intended interventions, (3) missing outcome data, (4) measurement of the outcome, and (5) selection of the reported result. The global evaluation of this tool can be as follows: low (if this score is obtained in the 5 domains); some concerns (if the score is obtained in one or more domains); and high (when this score is obtained in at least one domain). The RoB-2 score of each study is presented in Figure 2.

This evaluation was carried out by Mario Martinez-Torija and Víctor Serrano-Fernández The reliability between both evaluators was high, and any slight discrepancies were discussed between them until an agreement was reached. The opinion of the third observer (José Alberto Laredo-Aguilera) was considered in case of conflict between the principal investigators.

### 2.7. Data Analysis

A narrative synthesis was conducted, organised by outcome: cannulation success, pain, procedure time, nursing satisfaction, patient satisfaction, and complications. Study characteristics and individual findings were summarised in Table 3. Group-level summary statistics, effect estimates, confidence intervals, and *p* values were presented as reported in the original studies, where available. Findings were interpreted considering differences in study design, outcome definitions, intervention characteristics, and units of analysis. Patient-level results were distinguished from results based on repeated cannulations or sessions. No pooled estimates were calculated because the limited number of studies and differences in their units of analysis restricted the comparability of the available estimates.

### 2.8. Certainty of Evidence Assessment

The certainty of evidence for each assessed outcome was evaluated using the GRADE approach [33]. Evidence from randomized trials was initially rated as high and downgraded when concerns were identified regarding risk of bias, inconsistency, indirectness, imprecision, or publication bias. Inconsistency was assessed by comparing the direction and magnitude of study-level findings, considering differences in outcome definitions and measurement. Imprecision was assessed using the available study-level confidence intervals, sample sizes, and event counts, where applicable, while considering repeated observations within participants. No funnel plots or statistical tests for publication bias were used because of the small number of studies. Certainty assessments were based on the narrative synthesis rather than pooled estimates. The detailed GRADE assessments are provided in Supplementary Table S2 [28,29,30,31,32].

### 2.9. Deviations from the Registered Protocol

The original PROSPERO record (CRD42024613025) specified pain and cannulation time as the main outcomes, and patient satisfaction, nursing satisfaction, and cannulation success as additional outcomes. Cannulation-related complications were additionally assessed in the narrative synthesis. The findings were synthesized narratively, and the PROSPERO title and data synthesis methods were updated accordingly. GRADE assessments were also conducted, although this procedure was not specified in the original registration. During manuscript revision, the nursing filter was removed from the search strategies, CINAHL and Cochrane Library were added, and the searches and study selection process were repeated.

## 3. Results

### 3.1. Study Selection

The selection of studies was carried out as shown in the PRISMA flow chart (Figure 1), which details the study selection process from identification to final inclusion. Initially, 1210 articles were identified through the databases. After eliminating duplicates (*n* = 145), the titles and abstracts of the remaining 1065 studies were reviewed for their relevance according to the previously defined inclusion and exclusion criteria.

In the screening phase, 16 results were selected for complete reading, and those articles that did not meet the selection criteria (*n* = 11) were excluded. Finally, 5 RCTs were included [28,29,30,31,32] in the systematic review. The excluded studies [16,17,18,19,20,21,22,23,24,25,26] and their corresponding reasons for exclusion are detailed in Supplementary Table S1.

Among the ClinicalTrials.gov records screened, one randomized crossover study of ultrasound-guided, device-assisted needle insertion (NCT07856095) was listed as not yet recruiting, with no results available. A second potentially relevant randomized trial (NCT06381310) had an unknown recruitment status and no results posted. Neither provided outcome data for inclusion in the narrative synthesis.

### 3.2. Characteristics of the Selected Studies

After the search was conducted, 5 studies [28,29,30,31,32] that met the inclusion criteria to be included in this review were selected.

The five included trials [28,29,30,31,32] comprised 142 patients: 131 participants in four parallel-group trials (66 in the intervention groups and 65 in the control groups) and 11 participants in one crossover trial, in which each patient received both techniques. Operator characteristics, ultrasound training, equipment, guidance techniques, access selection criteria, and intervention duration or follow-up are summarized in Table S3 [28,29,30,31,32].

All the subjects were included in an HD program. Table 3 shows the characteristics of the included studies.

**Table 3:** Characteristics of the included studies.

Author, Year and Country	Design	Study Population	Intervention	RESULTS			Conclusions	Risk of Bias
				Pain	Time	Other Outcomes		
Kumbar et al. [28] 2018 United States	RCT	Subjects: 9 IG: 5 CG: 4	USG vs. traditional cannulation. Duration of 3 months	IG > CG (2.6 vs. 2.1; *p* > 0.05)	IG > CG (101.8 vs. 22.3 s; *p* < 0.001)	NSD in puncture success. USG cannulation allows to obtain better pump flows. NSD in patient satisfaction	USG cannulation is feasible and does not promote errors during channeling of access	Some concerns
Eves et al. [29] 2020 United Kingdom	RCT	Subjects: 32 IG: 16 CG: 16	USG vs. traditional cannulation. Duration of 4 weeks	IG < CG (1.85 vs. 2.27; *p* > 0.05)	IG > CG (303 vs. 230 s; *p* < 0.05)	Fewer attempts to cannulate with USG cannulation. Inconclusive results in staff satisfaction; NSD in patients	Despite requiring more time, USG cannulation can be used effectively in dialysis units	Some concerns
Chen et al. [30] 2022 Singapore	RCT	Subjects: 50 IG: 25 CG: 25	USG vs. traditional cannulation. Duration of 1 year	IG < CG (1.48 vs. 2.13; *p* < 0.05)	IG < CG (5.41 vs. 6.21 min; *p* > 0.05)	Higher success rate in IG. USG cannulation improves staff satisfaction after training	USG cannulation is associated with higher success rates. It is a safe practice that can be carried out in hospitals	Some concerns
Wu et al. [31] 2022 China	RCT	Subjects: 40 IG: 20 CG: 20	In-plane USG vs. conventional cannulation	VAS > 4: IG < CG (0.34% vs. 1.12%; *p* = 0.027)	Not reported	Higher first-attempt success and patient satisfaction in IG. Fewer hematomas and bleeding events in IG	USG cannulation showed favorable procedural and patient satisfaction outcomes	Some concerns
Schoch et al. [32] 2023 Australia	RCT	Subjects: 11 patients (crossover)	USG vs. traditional cannulation	Arterial pain: MD −0.5; *p* = 0.242). Venous pain: MD −0.4; *p* = 0.216).	IG > CG (8.42 vs. 6.73 min; *p* = 0.008)	Two miscannulations, both with traditional cannulation. Nurses’ acceptance improved over time	USG cannulation was feasible but required more time	Some concerns

CG: Control group. IG: Intervention group. USG: Ultrasound-guided. NSD: No significant difference.

### 3.3. Risk of Study Bias

Risk of bias for the pain outcome was assessed using RoB 2 (Figure 2). All five studies were judged to raise some concerns overall, and none were rated high risk of bias. Some concerns were identified in the randomization process (D1) for Kumbar et al. [28] and Wu et al. [31]; deviations from the intended interventions (D2) for Kumbar et al. [28]; missing outcome data (D3) for Eves et al. [29]; measurement of the outcome (D4) for all five studies; and selection of the reported result (D5) for Kumbar et al. [28] and Eves et al. [29]. The remaining domains were judged as low risk of bias.

### 3.4. Success in Cannulation

In two studies, differences were reported regarding the need for multiple attempts between the intervention and control groups. In the study carried out by Chen et al. [30]. the success rates were 96% and 72% when comparing USG and traditional punctures, respectively (*p* = 0.049). In this case, the total number of punctures on the AVF was considered.

On the other hand, the study carried out by Eves et al. [29] reported a lower mean number of attempts in those AVFs with difficult palpation and the presence of recent complications was lower for USG cannulation, with 3.08 vs. 5.05 and a *p* value < 0.001.

The study carried out by Kumbar et al. [28] found no significant differences between groups, with a mean of attempts of 1 in both groups (*p* = 0.64). However, the effective blood flow measured per session was greater in the intervention group than in the control group (314.4 ± 73.9 mL/min vs. 262.4 ± 59.0 mL/min; *p* < 0.001). Similar to the study by Chen et al. [30], the number of additional punctures was considered for each AVF puncture. The heterogeneity in the measurement of cannulation success across the studies made it impossible to carry out a meta-analysis.

Wu et al. [31] randomized 40 patients with upper-arm AVFs to ultrasound-guided in-plane cannulation or conventional cannulation, with 20 patients in each group. Both techniques were performed by the same experienced nurse. The reported first-attempt success rate was higher with ultrasound guidance than with conventional cannulation (98.71% vs. 88.27%; *p* < 0.001). These percentages were calculated from repeated cannulations rather than individual patients. However, the number of cannulations reported in the methods differed from that presented in the results tables, and the statistical analysis did not describe adjustment for repeated observations within patients.

Finally, Schoch et al. [32] reported two miscannulations among 252 needle cannulations, both occurring with conventional cannulation. The small number of events precluded further comparative analysis. These observations arose from repeated cannulations within patients and nurses and were therefore summarized narratively.

### 3.5. Pain Assessment

Kumbar et al. [28], Eves et al. [29], and Chen et al. [30] reported continuous pain scores. Chen et al. reported lower pain scores with ultrasound-guided cannulation (1.48 vs. 2.13; *p* = 0.012). Kumbar et al. reported higher scores with ultrasound guidance, although the difference was not statistically significant (2.6 vs. 2.1; *p* = 0.12). Eves et al. reported lower scores with ultrasound guidance in the subgroup described as having greater cannulation difficulty, but the difference was not statistically significant (1.85 vs. 2.27; *p* = 0.498). Comparisons stratified by fistula diameter were also not statistically significant.

Wu et al. [31] assessed pain using a visual analogue scale but reported the frequency of scores > 4 during hemodialysis rather than mean pain scores. According to the results table, this outcome occurred in 4 of 1168 cannulations in the ultrasound-guided group (0.34%) and 13 of 1159 cannulations in the conventional group (1.12%; *p* = 0.027). This study was included in the narrative synthesis. Furthermore, the reported assessment during hemodialysis was not directly equivalent to pain measured at the time of cannulation.

Schoch et al. [32] assessed arterial and venous needle insertion pain separately on a 0–10 scale. Adjusted mixed models showed no statistically significant differences between ultrasound-guided and conventional cannulation for arterial pain (MD −0.5; 95% CI −1.4–0.4; *p* = 0.242) or venous pain (MD − 0.4; 95% CI − 1.2–0.3; *p* = 0.216). These findings were retained in the narrative synthesis.

### 3.6. Time Spent

Among the three parallel-group trials reporting procedure time, Chen et al. [30] reported a shorter duration with ultrasound guidance, although the difference was not statistically significant (5.41 vs. 6.21 min; *p* = 0.057). Eves et al. [29] and Kumbar et al. [28] reported longer durations with ultrasound guidance (303 vs. 230 s, *p* < 0.05, and 101.8 vs. 22.3 s, *p* < 0.001, respectively). In the crossover trial by Schoch et al. [32], procedure time was 1.69 min longer with ultrasound guidance (95% CI 0.65–2.77; *p* = 0.008). Wu et al. [31] did not report this outcome. Differences in the timed intervals, study designs, access characteristics, and units of analysis limit direct comparison.

### 3.7. Patient Satisfaction

Four of the five included trials reported patient satisfaction or comfort outcomes [28,29,31,32]. The first study [28] reported that 94.6% of the USG cannulations were very comfortable, compared to 82.0% for the traditional method (*p* = 0.06). Furthermore, none of the USG cannulations were rated as indifferent or uncomfortable, whereas 8% of the control group reported such assessments. In contrast, the study by Eves et al. [29] found no differences in comfort (*p* = 0.98) or anxiety (*p* = 0.87) between the intervention and control group.

Wu et al. [31] assessed patient satisfaction after each hemodialysis session using a study-specific questionnaire covering the nurse’s cannulation technique, the acceptability of cannulation pain, and satisfaction with the dialysis process. Satisfaction ratings were higher in the ultrasound-guided group than in the conventional group (*p* < 0.001). However, the questionnaire was not reported as validated, and the analysis used repeated assessments from the same patients without describing adjustment for within-patient dependence. In Schoch et al. [32], patients reported being “extremely satisfied” or “somewhat satisfied” in 97.6% of assessments, with no difference detected between ultrasound-guided and conventional cannulation.

### 3.8. Nursing Satisfaction

Eves et al. [29] reported mixed results regarding the level of satisfaction among nursing staff. Approximately 75% of the staff perceived a greater need for the time spent on the technique. On the other hand, they recognize that greater use and experience in USG cannulation increases the confidence of staff, which is why training in this technique is advocated. However, a correlation was also found between greater experience of the personnel in the service with less predisposition to training in USG cannulation.

Regarding the study carried out by Chen et al. [30], differences were only described in the assessment of staff satisfaction following USG puncture training. Statistically significant differences were observed in the satisfaction scores before and after the training (26.6 vs. 36.4 points, respectively; *p* = 0.014).

In Schoch et al. [32], nurse surveys and interviews indicated increasing acceptance of ultrasound-guided cannulation over time, including perceived ease of use, suitability, and willingness to use it when available. Nurses did not recommend ultrasound guidance for every cannulation. These findings describe acceptability over time rather than a randomized comparison of satisfaction between techniques.

### 3.9. Complications

The trials reported extravasation, hematoma, bleeding, needle thrombosis, infection, needle replacement or reinsertion, and vascular access dysfunction [28,29,30].

Reported complications included extravasation, hematoma, bleeding, needle thrombosis, infection, needle replacement or reinsertion, and vascular access dysfunction [28,29,30,31]. The trials by Kumbar et al. [28], Eves et al. [29], and Chen et al. [30] did not demonstrate statistically significant differences in the reported complication outcomes between ultrasound-guided and conventional cannulation.

In contrast, Wu et al. [31] reported lower frequencies of hematoma and bleeding with ultrasound guidance. According to the results table, hematoma occurred in 4 of 1168 cannulations in the ultrasound-guided group (0.34%) and 94 of 1159 cannulations in the conventional group (8.11%; *p* < 0.001). Bleeding occurred in 7 of 1168 ultrasound-guided cannulations (0.59%) and 26 of 1159 conventional cannulations (2.24%; *p* = 0.001). These findings require caution because the denominators differed between the methods and results sections, and the analysis did not describe adjustment for repeated cannulations within patients.

The study performed by Chen et al. [30] is the only one that presents aggregated results, showing 8% of the patients in the intervention group experienced complications, compared to 16% in the control group (*p* = 0.667). Similarly, the studies by Kumbar et al. [28] and Eves et al. [29] did not show any differences in the previously described categories of complications.

Finally, Schoch et al. [32] reported two miscannulations, both with conventional cannulation, but provided insufficient events for a comparative analysis of this outcome.

### 3.10. Certainty of Evidence

The certainty of evidence was low for all assessed outcomes. Table 4 summarizes the findings and certainty assessments for each outcome. Results were synthesized narratively, without pooled effect estimates. Detailed GRADE assessments and explanations are provided in Supplementary Table S2.

## 4. Discussion

This systematic review identified five small randomized trials evaluating ultrasound-guided AVF cannulation. Some trials reported improved cannulation success or lower pain, but the findings did not establish consistent benefits across settings and outcome measures. Effects on complications and patient satisfaction remain uncertain, while staff findings mainly describe confidence and acceptability. Three of the four trials reporting procedure time found longer durations with ultrasound guidance. These findings require caution because of low-certainty evidence and differences in populations, interventions, outcome definitions, and units of analysis.

Three of the five included trials reported improvements in cannulation success or fewer cannulation attempts with USG cannulation [29,30,31]. These findings are consistent with previous research on ultrasound-guided CVC insertion [34,35], peripheral venous cannulation in difficult-access situations [36,37], and arterial access in critically ill patients [38,39]. However, the findings of Wu et al. [31] require caution because the analysis did not describe adjustment for repeated cannulations within patients, and the cannulation totals differed between the methods and results tables.

Previous nursing training plays an important role in success rates. In one of the studies that reported differences [29], the nurses’ experience in USG techniques ranged from 6 months to 20 years. In another study [30], which also reported differences in success rates, nurses received both theoretical and practical training from an interventional nephrologist. Kumbar et al. [28], who found no significant difference in cannulation success, provided 3 h of training to the nursing staff. Schoch et al. [32] reported two miscannulations, both with conventional cannulation, but the small number of events precluded a comparative analysis. Outside the randomized evidence, a single-centre quality-improvement study evaluated ultrasound-guided cannulation in relation to AVF infiltration rates [40]. These findings may inform feasibility but do not establish comparative effectiveness.

The potential benefit of USG cannulation may depend on access characteristics and operator experience. Trials involving difficult-to-cannulate AVFs reported improvements in cannulation outcomes [29,30]. In contrast, Schoch et al. [32] studied established AVFs and observed few miscannulations, limiting the ability to detect a difference between techniques. These findings do not establish that ultrasound guidance is necessary for every cannulation or that it improves long-term AVF survival. Access maturity may also shape cannulation difficulty; early cannulation of native AVFs represents a distinct clinical context [41]. More broadly, AVF complications can threaten access function and survival [42], although the included trials do not establish that ultrasound guidance reduces cannulation-related complications.

Findings concerning pain varied across trials. Chen et al. [30] reported lower insertion pain scores with ultrasound guidance, whereas Kumbar et al. [28], Eves et al. [29], and Schoch et al. [32] did not demonstrate clear reductions. Wu et al. [31] reported fewer episodes of pain during hemodialysis, which is not directly equivalent to insertion pain. Differences in assessment timing, outcome measures, and analyses of repeated observations limit comparison. The evidence does not establish a consistent reduction in pain.

Ultrasound-guided procedures in other settings, including peripheral venous access and other needle-based interventions, have also been studied [43,44,45,46,47,48]. These findings provide only indirect context and cannot be assumed to apply to repeated AVF cannulation because of differences in anatomy, indication, and technique. A report specifically addressing ultrasound use in difficult-to-cannulate AVFs offers additional context, but does not replace randomized comparative evidence [49].

Three of the four trials reporting procedure duration found longer times with ultrasound guidance [28,29,32], whereas Chen et al. [30] reported a shorter duration. Wu et al. [31] did not assess this outcome. Differences in the timed interval, access characteristics, and operator training limit direct comparison. The low-certainty evidence does not support concluding that ultrasound guidance can be introduced without additional procedure time.

Patient and nursing satisfaction were assessed in several included trials, although the measures and findings varied. Wu et al. [31] reported higher patient satisfaction ratings with USG cannulation but used a study-specific questionnaire without reported validation and analyzed repeated evaluations without describing adjustment for within-patient dependence. In contrast, Schoch et al. [32] reported high patient satisfaction with both techniques without a detected difference between them. A study by Xi et al. [50] states that one of the main reasons for patient dissatisfaction is the multiple attempts derived from the first punctures, as well as the fear of pain, which leads to avoid choosing AVF as the preferred VA. Whether ultrasound-guided cannulation influences patients’ vascular access preferences remains uncertain.

Training may be relevant to the implementation of ultrasound-guided cannulation. Niyyar et al. [51] described an ultrasound-based simulation protocol for cannulation in outpatient hemodialysis units, while Harwood et al. [52] discussed factors associated with AVF cannulation success. Studies in other vascular-access settings have also addressed ultrasound-guided cannulation and training [53,54,55], although their findings may not be directly applicable to AVF cannulation. In the included trial by Schoch et al. [32], nurses’ acceptance of ultrasound-guided cannulation increased over time, although they did not recommend its use for every cannulation. The available evidence does not establish the optimal training approach.

### 4.1. Limitations and Strengths

The present review has several limitations. Five RCTs were included in the narrative synthesis [28,29,30,31,32]. The small number of trials and differences in outcome definitions, reporting, study designs, and units of analysis precluded meta-analysis. In Wu et al. [31], inconsistent cannulation totals and the lack of a described adjustment for repeated observations introduced uncertainty. Schoch et al. [32] used a crossover design with repeated observations within patients and nurses. Pain results were reported separately for arterial and venous needle insertion, and condition-specific standard deviations for procedure time were unavailable. Potential period or carryover effects in the crossover design also require consideration. Subjective outcomes such as pain and satisfaction may be influenced by knowledge of the intervention received.

The review has several strengths. It focuses on randomized trials directly relevant to nursing practice in hemodialysis units and considers both procedural outcomes and patient-reported experiences. The expanded searches and reassessment of eligibility added two trials to the narrative synthesis, including a randomized crossover trial. Risk-of-bias assessment using RoB 2 and outcome-specific certainty assessment using GRADE support the interpretation of the findings. Studies with incompatible outcome reporting were retained in the narrative synthesis, allowing their findings to be considered without combining non-comparable data.

### 4.2. Implications for Clinical Practice

Ultrasound guidance may be considered for selected patients, particularly those with difficult-to-cannulate AVFs. However, the available evidence does not support its routine use for every cannulation or its designation as the standard or preferred technique. Implementation should consider operator training, competency assessment, equipment availability, and the potential for longer procedure times. Improvements in complications, satisfaction, and long-term AVF function have not been established.

## 5. Conclusions

The effects of ultrasound-guided cannulation on pain varied across trials. The effect on procedure time remains uncertain, and the crossover trial reported longer cannulation time with ultrasound guidance. Evidence concerning cannulation success, complications, and patient or nursing satisfaction remains limited and inconsistent. Larger randomized trials with clearly defined outcomes and analyses that account for repeated observations are needed to determine which patients benefit most.

## Figures and Tables

**Table 1:** PICO question.

Population	Intervention	Control	Outcome
Patients with AVF in HD program	USG AVF puncture	Standard AVF puncture	Decreased pain and complications in the patient, decreased cannulation time, increased success rate, and increased patient and nursing satisfaction

**Table 2:** Search strategies.

Database	Search String
PubMed	(echography OR ultrasound OR ultrasonography) AND (punction OR cannulation OR needle) AND hemodialysis
Scopus	TITLE-ABS ((echography OR ultrasound OR ultrasonography) AND (punction OR cannulation OR needle) AND hemodialysis)
ClinicalTrials	(echography OR ultrasound OR ultrasonography) AND (punction OR cannulation OR needle) AND hemodialysis
Web of Science	TS = ((echography OR ultrasound OR ultraso-nography) AND (punction OR cannulation OR needle) AND hemodialysis)
CINAHL	(echography OR ultrasound OR ultraso-nography) AND (punction OR cannulation OR needle) AND hemodialysis
Cochrane Library	((echography OR ultrasound OR ultraso-nography) AND (punction OR cannulation OR needle) AND hemodialysis): ti, ab, kw

**Table 4:** Summary of findings for ultrasound-guided versus conventional arteriovenous fistula cannulation in patients undergoing hemodialysis.

**Outcome**	**Evidence Base**	**Summary of Findings**	**Certainty of Evidence (GRADE)**
Cannulation success	142 patients (5 trials)	Ultrasound guidance may improve first-attempt success or reduce cannulation attempts in some settings. Definitions of success and units of analysis differed across studies.	Low
Pain	142 patients (5 trials)	Effects on pain varied. Some studies reported lower insertion pain scores or fewer episodes of pain during hemodialysis, while others found small differences in either direction. Assessment timing and outcome measures differed.	Low
Cannulation time	102 patients (4 trials)	Three trials reported longer procedure times with ultrasound guidance, while one reported a shorter duration. Differences in the timed interval limited comparison.	Low
Patient satisfaction	92 patients (4 trials)	Findings did not establish a consistent improvement in satisfaction or comfort. Measures varied, and some analyses used repeated assessments without a described adjustment for within-patient dependence.	Low
Nursing satisfaction	Number of nurses not reported (3 studies)	Staff reports suggested greater confidence or acceptance with training and experience, alongside concerns about additional procedure time. These findings do not establish a comparative effect on nursing satisfaction.	Low
Complications	142 patients (5 trials)	Some studies reported fewer hematoma and bleeding events, while others provided limited comparative evidence. Different complication definitions and analyses of repeated procedures limited interpretation.	Low

Patient totals refer to enrolled participants, not outcome observations: Kumbar et al., 9 patients [28]; Eves et al., 32 [29]; Chen et al., 50 [30]; Wu et al., 40 [31]; and Schoch et al., 11 [32]. Some outcomes were analysed using repeated cannulation events in Kumbar et al. [28], Eves et al. [29], and Wu et al. [31], and repeated nurse–patient encounters in Schoch et al. [32]; Chen et al. [30] reported results by randomized patient. Wu et al. reported discrepant cannulation totals in the Methods and Results [31]; denominators were not pooled across studies.

## Data Availability

No new data were created or analyzed in this study. Data sharing is not applicable to this article.

## Supplementary Materials

The following supporting information can be downloaded at https://www.mdpi.com/article/10.3390/healthcare14213577/s1, Table S1: Excluded studies; Table S2: Certainty of evidence (GRADEpro); Table S3: Operator, training, equipment, and intervention characteristics of the included trials.

## Abbreviations

The following abbreviations are used in this manuscript:

AVF	Arteriovenous Fistula
CG	Control Group
CI	Confidence Interval
CVC	Central Venous Catheter
GDT	Guideline Development Tool
GRADE	Grading of Recommendations Assessment, Development and Evaluation
HD	Hemodialysis
IG	Intervention Group
MD	Mean Difference
NSD	No Significant Difference
PICO	Population, Intervention, Control, And Outcome
PRISMA	Preferred Reporting Items for Systematic Reviews and Meta-Analyses
PROSPERO	International Prospective Register of Systematic Reviews
RCT	Randomized Controlled Trial
RevMan	Review Manager
RoB 2	Risk Of Bias 2
SD	Standard Deviation
