# Supplementary material for: Ultrasound-Guided Cannulation of Arteriovenous Fistulas in Hemodialysis Units: A Systematic Review
Source: Healthcare (Basel). 2026 Oct 22;14(21):3577. doi: 10.3390/healthcare14213577 (PMC13762872; doi:10.3390/healthcare14213577)
Supplement: Supplementary file 1 [file healthcare-14-03577-s001.zip › healthcare-4594884-supplementary.pdf]

Section and Topic	Item #	Checklist item	Location where item is reported
**TITLE**			
Title	1	Identify the report as a systematic review.	Page 1
**ABSTRACT**			
Abstract	2	See the PRISMA 2020 for Abstracts checklist.	Page 1-2
**INTRODUCTION**			
Rationale	3	Describe the rationale for the review in the context of existing knowledge.	Page 2-3
Objectives	4	Provide an explicit statement of the objective(s) or question(s) the review addresses.	Page 2-3
**METHODS**			
Eligibility criteria	5	Specify the inclusion and exclusion criteria for the review and how studies were grouped for the syntheses.	Pages 3-4
Information sources	6	Specify all databases, registers, websites, organisations, reference lists and other sources searched or consulted to identify studies. Specify the date when each source was last searched or consulted.	Pages 3-4
Search strategy	7	Present the full search strategies for all databases, registers and websites, including any filters and limits used.	Page 3
Selection process	8	Specify the methods used to decide whether a study met the inclusion criteria of the review, including how many reviewers screened each record and each report retrieved, whether they worked independently, and if applicable, details of automation tools used in the process.	Page 4
Data collection process	9	Specify the methods used to collect data from reports, including how many reviewers collected data from each report, whether they worked independently, any processes for obtaining or confirming data from study investigators, and if applicable, details of automation tools used in the process.	Page 5
Data items	10a	List and define all outcomes for which data were sought. Specify whether all results that were compatible with each outcome domain in each study were sought (e.g. for all measures, time points, analyses), and if not, the methods used to decide which results to collect.	Pages 3 and 5, Table 1
	10b	List and define all other variables for which data were sought (e.g. participant and intervention characteristics, funding sources). Describe any assumptions made about any missing or unclear information.	Pages 5 and 7
Study risk of bias assessment	11	Specify the methods used to assess risk of bias in the included studies, including details of the tool(s) used, how many reviewers assessed each study and whether they worked independently, and if applicable, details of automation tools used in the process.	Page 5
Effect measures	12	Specify for each outcome the effect measure(s) (e.g. risk ratio, mean difference) used in the synthesis or presentation of results.	Page 5

Section and Topic	Item #	Checklist item	Location where item is reported
Synthesis methods	13a	Describe the processes used to decide which studies were eligible for each synthesis (e.g. tabulating the study intervention characteristics and comparing against the planned groups for each synthesis (item #5)).	Not applicable
	13b	Describe any methods required to prepare the data for presentation or synthesis, such as handling of missing summary statistics, or data conversions.	Page 5
	13c	Describe any methods used to tabulate or visually display results of individual studies and syntheses.	Page 5; Tables 3 and 4
	13d	Describe any methods used to synthesize results and provide a rationale for the choice(s). If meta-analysis was performed, describe the model(s), method(s) to identify the presence and extent of statistical heterogeneity, and software package(s) used.	Page 5
	13e	Describe any methods used to explore possible causes of heterogeneity among study results (e.g. subgroup analysis, meta-regression).	NA
	13f	Describe any sensitivity analyses conducted to assess robustness of the synthesized results.	Not applicable
Reporting bias assessment	14	Describe any methods used to assess risk of bias due to missing results in a synthesis (arising from reporting biases).	Page 5
Certainty assessment	15	Describe any methods used to assess certainty (or confidence) in the body of evidence for an outcome.	Page 6
**RESULTS**			
Study selection	16a	Describe the results of the search and selection process, from the number of records identified in the search to the number of studies included in the review, ideally using a flow diagram.	Pages 4 and 6, Figure 1
	16b	Cite studies that might appear to meet the inclusion criteria, but which were excluded, and explain why they were excluded.	Page 6
Study characteristics	17	Cite each included study and present its characteristics.	Pages 6-7 and Table 3
Risk of bias in studies	18	Present assessments of risk of bias for each included study.	Pages 5 and 7, Figure 2
Results of individual studies	19	For all outcomes, present, for each study: (a) summary statistics for each group (where appropriate) and (b) an effect estimate and its precision (e.g. confidence/credible interval), ideally using structured tables or plots.	Page 7, Table 3
Results of syntheses	20a	For each synthesis, briefly summarise the characteristics and risk of bias among contributing studies.	Pages 5-7, Figure 2, Table 3
	20b	Present results of all statistical syntheses conducted. If meta-analysis was done, present for each the summary estimate and its precision (e.g. confidence/credible interval) and measures of statistical heterogeneity. If comparing groups, describe the direction	Not applicable

Section and Topic	Item #	Checklist item	Location where item is reported
		of the effect.	
	20c	Present results of all investigations of possible causes of heterogeneity among study results.	Not applicable
	20d	Present results of all sensitivity analyses conducted to assess the robustness of the synthesized results.	Not applicable
Reporting biases	21	Present assessments of risk of bias due to missing results (arising from reporting biases) for each synthesis assessed.	Not assessed (<10 studies)
Certainty of evidence	22	Present assessments of certainty (or confidence) in the body of evidence for each outcome assessed.	Pages 10,11; Table 4 and Table S2
**DISCUSSION**			
Discussion	23a	Provide a general interpretation of the results in the context of other evidence.	Pages 10-11
	23b	Discuss any limitations of the evidence included in the review.	Page 12
	23c	Discuss any limitations of the review processes used.	Page 12
	23d	Discuss implications of the results for practice, policy, and future research.	Page 12
**OTHER INFORMATION**			
Registration and protocol	24a	Provide registration information for the review, including register name and registration number, or state that the review was not registered.	Page 3
	24b	Indicate where the review protocol can be accessed, or state that a protocol was not prepared.	Page 3
	24c	Describe and explain any amendments to information provided at registration or in the protocol.	Page 3
Support	25	Describe sources of financial or non-financial support for the review, and the role of the funders or sponsors in the review.	Page 13
Competing interests	26	Declare any competing interests of review authors.	Page 13
Availability of data, code and other materials	27	Report which of the following are publicly available and where they can be found: template data collection forms; data extracted from included studies; data used for all analyses; analytic code; any other materials used in the review.	Page 13

Table S1. Excluded studies [16-26]

First author	Title	Reason for exclusion
Thajudeen B, et al. [16] 2020	Comparative study between ultrasound-guided arteriovenous fistula cannulation versus conventional method in adult hemodialysis patients	Conference abstract
Ibrik O, et al. [17] 2000	Ultrasound-guided cannulation versus the landmark-guided technique for hemodialysis vascular access	Non AVF punction
Boudet J, et al. [18] 2025	Assessment of ultrasound-guided arteriovenous fistula cannulation in pediatric hemodialysis	Pediatric population
Ward F, et al. [19] 2017	Ultrasound-Guided Cannulation of the Hemodialysis Arteriovenous Access	Review article
Khanoussi A, et al. [20] 2022	Nurses' Use of Ultrasound Location for Puncturing Arteriovenous Fistulas in Hemodialysis	Non-randomized design
Schoch M, et al. [21] 2015	Utilising point of care ultrasound for vascular access in haemodialysis	Continuing professional development article
Patel R, et al. [22] 2015	Bedside Ultrasonography for Arteriovenous Fistula Cannulation	Case report
Thompson P, et al. [23] 2026	Feasibility study of ultrasound-guided, power-assisted needle insertion for hemodialysis cannulation using an innovative, handheld device	Non-randomized design
Kamata T, et al. [24] 2016	Ultrasoundguided cannulation of hemodialysis access	Review article
Luehr A. [25] 2018	Use of Ultrasound Guidance During Cannulation of Arteriovenous Fistulas	Non-randomized design
Nalesso F, et al. [26] 2020	Ultrasound for the Clinical Management of Vascular Access Cannulation and Needle Position in Hemodialysis Patients	Non-randomized design

Table S2. Certainty of evidence (GRADEpro) [28-32]

Certainty assessment							Impact	Certainty	Importance
№ of studies	Study design	Risk of bias	Inconsistency	Indirectness	Imprecision	Other considerations			
**Success in Cannulation**									
5 [28-32]	randomised trials	serious[a]	not serious	not serious	serious[a]	none	Ultrasound guidance may improve first-attempt success or reduce cannulation attempts in some settings. Findings varied across definitions of success, and several estimates were based on repeated procedures. No pooled estimate was calculated.	⊕⊕◯◯ Low[a,b]	
**Pain**									
5 [28-32]	randomised trials	serious[a]	not serious	not serious	serious[b]	none	Ultrasound guidance showed variable effects on pain. Some studies reported lower insertion pain scores or fewer episodes of pain during hemodialysis, whereas others found small differences in either direction. Differences in assessment timing, outcome measures, and units of analysis limited comparison. No pooled estimate was calculated.	⊕⊕◯◯ Low[a,b]	
**Time**									
4 [28-30,32]	randomised trials	serious[a]	not serious	not serious	serious[b]	none	Three trials reported longer procedure times with ultrasound guidance, whereas Chen et al. reported a shorter duration. Schoch et al. reported an increase of 1.69 minutes (95% CI 0.65–2.77). Differences in the timed interval limit direct comparison.	⊕⊕◯◯ Low[a,b]	
**Patient satisfaction**									
4 [28-30,32]	randomised trials	serious[a]	not serious	not serious	serious[b]	none	Findings did not establish a consistent improvement in patient satisfaction or comfort. Measures varied across studies, and some analyses used repeated assessments without a described adjustment for within-patient dependence. No pooled estimate was calculated.	⊕⊕◯◯ Low[a,b]	
**Nursing satisfaction**									

Certainty assessment							Impact	Certainty	Importance
№ of studies	Study design	Risk of bias	Inconsistency	Indirectness	Imprecision	Other considerations			
3 [29,30,32]	randomised trials	serious[a]	not serious	not serious	serious[b]	none	Staff reports suggested increasing confidence or acceptance with training and experience, alongside concerns about additional procedure time. These findings do not establish a comparative effect of ultrasound guidance on nursing satisfaction.	⨁⨁◯◯ Low[a,b]	
**Complications**									
5 [28-32]	randomised trials	serious[a]	not serious	not serious	serious[b]	none	The effect on cannulation-related complications remains uncertain. Some studies reported fewer hematoma and bleeding events, while others provided limited comparative evidence. Differences in outcome definitions and analyses of repeated procedures limited interpretation. No pooled estimate was calculated.	⨁⨁◯◯ Low[a,b]	

CI: confidence interval

Explanations

a. Some studies did not describe adjustment for repeated observations within patients, which may have overstated the precision of their results.

b. Small patient samples and incomplete confidence intervals limited assessment of clinically important benefits or harm.

Table S3. Operator, training, equipment, and intervention characteristics of the included trials. [28-32]

Study	Operator profession and experience	Ultrasound training	Ultrasound device	Static/dynamic technique	AVF selection	Intervention duration / follow-up
**Kumbar et al. [28] 2018**	Four dialysis personnel performed intervention cannulations. Their professional qualifications were not specified. Control cannulations were performed by the specialist cannulation team; trained intervention personnel could also cannulate controls.	Up to 3 hours of simulated device training, followed by competency assessment.	Sonic Window handheld coronal-mode ultrasound device (Analogic Ultrasound).	Ultrasound assessment and site identification before cannulation. Real-time guidance was optional, at the operator's discretion.	New AVFs cleared for initial cannulation by vascular surgery following physical examination.	Intervention: 3 weeks or 9 dialysis sessions, whichever occurred first. Follow-up: 3 months.
**Eves et al. [29] 2020**	Trained dialysis staff; the access classification specified nursing staff for difficult AVFs, with seniority matched to cannulation difficulty.	Two theory sessions and two practical sessions using phantom models; competency assessment requiring at least three independent successful cannulations; supervised patient practice over 4 weeks before the trial.	Sonosite Edge II with HFL50X straight probe.	Dynamic ultrasound guidance using transverse imaging. Rope-ladder cannulation.	Difficult-to-cannulate AVFs classified as amber, red, or black; adults receiving three weekly dialysis sessions with two needles. Active or recent complications within 2 weeks were excluded.	Cannulation events randomized over 4 weeks.
**Chen et al. [30] 2022**	Renal nurses. Ten entered training; five achieved competency certification before performing ultrasound-guided cannulation.	Training by accredited interventional nephrologists, followed by competency assessment. Training duration was not specified in the main report.	Handheld ultrasound with tablet and probe; manufacturer and model were not	Dynamic guidance with transverse probe placement and continuous visualization during needle advancement.	Difficult accesses, including first cannulation, previous failed cannulation, postintervention accesses, partial stenosis, bruising/hematoma, clots, or deep accesses. Accesses with caliber ≤4	Study conducted January 2021–January 2022. Individual follow-up duration was not clearly specified.

Study	Operator profession and experience	Ultrasound training	Ultrasound device	Static/dynamic technique	AVF selection	Intervention duration / follow-up
			specified in the main report.		mm or depth ≥8 mm were excluded and cannulated by physicians.	
**Wu et al. [31] 2022**	The same experienced nurse performed cannulation in both groups.	Ultrasound-specific training was not reported.	Mindray M7 Super portable color Doppler ultrasound with L12-4s probe.	Dynamic, longitudinal in-plane guidance, with visualization of the needle trajectory and adjustment during insertion.	Upper-arm AVFs 6–26 weeks after creation; vessel diameter ≥4 mm, depth 4–7 mm, and flow >600 mL/min. Patients with diabetes or chronic vasculitis were excluded.	Recruitment January 2020–December 2021. Individual follow-up duration was not clearly specified.
**Schoch et al. [32] 2023**	Registered nurses with formal hemodialysis training; nine recruited and seven completed the study. Among completers, median renal nursing experience was 12 years.	A 30-minute phantom training session with checklist assessment. Previous training varied, including up to 3 hours or a 2-day intensive workshop.	Terason uSmart 3200T; probe model was not specified.	Dynamic POCUS-guided cannulation; all nurses used longitudinal imaging. Static images were subsequently obtained in both conditions to assess needle position. Rope-ladder cannulation.	Adults with native AVFs cannulated with metal needles for >2 months. AVGs and plastic cannulas were excluded. No requirement for difficult cannulation.	Random-order crossover within nurse–patient dyads. Data collection: May 2018–December 2019; individual follow-up duration was not clearly specified.
